# Dataset for stability of high biomass and yield in maize under normal and intercropping conditions based on biplot, genotype stability index and land equivalent ratio

**DOI:** 10.1016/j.dib.2024.111161

**Published:** 2024-11-20

**Authors:** Mansyur, D. Ruswandi

**Affiliations:** a Faculty of Animal Husbandry, Universitas Padjadjaran, Bandung 45363, West Java, Indonesia; b Faculty of Agriculture, Universitas Padjadjaran, Bandung 45363, West Java, Indonesia

**Keywords:** Ammi, Biomass, Field corns, GGE biplot, Yield

## Abstract

Research on high-yielding and biomass of maize hibrids which adaptive to intercropping environment is important in the context of modern agriculture faced with the challenges of climate change. The field evaluation was conducted in Arjasari, West Java, Indonesia, for two seasons in three different cropping systems, namely: maize sole cropping, maize+soybean intercropping and maize+sweet potato intercropping. The evaluation applied a randomized completed block design with three replications. The article provides a data set of Combined Anova Table and biplot graphic of GGE and AMMI. Combined of Anova Table is provided to identify the effect of genotype, environment and their interaction for the traits observed. Thus biplot of GGE and AMMI is provided to identify representative environment and mega-environment for testing and development of hybrid maize; and to evaluate their adaptability in sole cropping as well as in intercropping with soybean and sweetpotato. The data in this article can be utilized by farmers to choose specific or stable maize hybrids for different cropping system.

Specifications Table

**Table:** 

Subject	*Data Article (Agricultural and Biological Science)*
Specific subject area	*Agronomy and Crop Science*
Data format	Analyzed data
Type of data	Table and Figure
Data collection	Raw data were taken from observation and measurement in the field. Data collection was done by measuring all plant in each experimental plot. Each plot composed of 60 plants for sole-cropping and 30 for intercropping. The yield in each plot was converted into tons ha-^1^. The data gathered were vegetative and yield traits. The traits evaluated were plant weight and cob weight with husks (CWH) for biomass and grain yield (GY) for yield. Data collection has been adjusted to match the maize descriptor.
Data source location	Samples were taken from 1 location at the Sarana Penelitian Latihan dan Pengembangan Pertanian (SPLPP) Universitas Padjadjaran in Arjasari District, Bandung Regency, West Java, Indonesia. Latitude 7.06112 S, longitude 107.64645 E, and altitude 950 m asl.
Data accessibility	Repository name: Mendeley Data Data identification number: DOI: 10.17632/vpschhv5kg.1 Direct URL to data: https://data.mendeley.com/datasets/vpschhv5kg/1
Related research article	–

## 1 Value of the Data

• The data in this article can be utilized by farmers to choose specific or stable maize hybrids for different cropping system.
• This data can help researchers, farmers, and industries to select ideal cropping system for breeding maize hybrids in different cropping systems.
• This dataset can be used in different types of research focusing on maize management, from agricultural, germplasm improvement, and environmental perspectives.

## 2 Background

The development of special-purpose maize requires a continuous and consistent selection process. Selection is an important stage in the genetic improvement of corn plants [1]. Simultaneous selection in a broad environment needs to be done to obtain genotypes that are adaptive, stable and high yielding. This selection can be done by utilizing stability analysis. Stability analysis can explain the effect of G x E interaction in detail. This analysis can also be used to evaluate the adaptability and yield stability of genotypes, which can help them in the difficult task of finding superior genotypes in the context of significant *G* × *E* interactions [2].

In addition to genetic stability, analysis is also needed to determine whether the cropping system applied is profitable. Land Equivalent Ratio (LER) values for different cropping systems are used to determine if a cropping system is profitable. LER analysis aims to determine the level of land productivity of intercropping applied compared to the sole cropping system [3]. A LER of more than 1 reveals the economic advantage of intercropping [4]. In addition to this analysis, satellite technology and machine learning models, are possible to predict land productivity and map cropping intensity [5,6]. Intercropping has been widely applied in various scales of agriculture and proven to improve resource use efficiency, increase agricultural productivity, reduce business risk, and reduce negative external influences compared to sole cropping [7]. The objectives of this study were to identify the effect of genotypes, environments, and their interactions (GEIs) on hybrid maize biomass and yield; identify representative environments and mega-environments for testing and development; and evaluate the adaptability of hybrid maize hybrids developed from crosses of elite strains with broad and specific adaptation in three maize cultivation systems in West Java, Indonesia.

## 3 Data Description

The combined analysis of variance (ANOVA) for hybrid maize evaluated at two seasons and three different cropping systems in Arjasari with the response variables biomass (CWH) and yield (GY) are presented in Table 1. The results of the combined analysis of variance on the tested characters showed that in the tested CWH and GY characters, almost all response variables of hybrid (G), environment (S, C and S × C), and their interaction (*G* × *S*, G × C, and *G* × *S* × C) had a very significant effect (*p* < 0.01) except for the CWH character which did not show a significant effect (*p* > 0.05) on the season (S) and environment (S × C) variables. The results of the combined ANOVA for the two characters tested showed that the coefficient of variance was 19.87 % in CWH and 15.02 % in GY.

**Table 1:** Combined ANOVA for CWH and GY of 24 maize hybrids under two seasons.

Sources	Df	CWH			GY		
		SS		%SS	SS		%SS
Rep	12	24.74		0.48 %	3.6		0.18 %
Season (S)	1	3.83		0.07 %	106.19	**	5.26 %
Cropping System (C)	2	3514.60	**	68.79 %	1412.49	**	70.00 %
S × C	2	0.85		0.02 %	68.44	**	3.39 %
Genotype (G)	23	285.1	**	5.58 %	37.78	**	1.87 %
*G* × *S*	23	141.38	**	2.77 %	66.81	**	3.31 %
G × C	46	317.3	**	6.21 %	94.27	**	4.67 %
GEIs (G × C × S)	46	220.04	**	4.31 %	109.82	**	5.44 %
Error	276	601.38		11.77 %	118.41		5.87 %
Total	431	5109.21		100.00 %	2017.81	**	100.00 %
Mean (t.ha^−1^)	7.43	4.36					
Min (t.ha^−1^)	2.57	1.44					
Max (t.ha^−1^)	21.11	14.15					
CV (%)	19.87	15.02 %					
SD	3.44	2.16					

SD = standard deviation; CV = Coefficient of variation; df = degree freedom; SS = Sum of square; MS = mean of square; ** *p* < 0.01.

Biomass (CWH) and yield (GY) for the hybrids tested in two seasons and three different cropping systems averaged 7.43 t.ha^−1^ and 4.36 t.ha^−1^, and the minimum values were 2.57 t.ha^−1^ and 1.44 t.ha^−1^, while the maximum values were 21.11 t.ha^−1^ and 14.15 t.ha^−1^ (Table 3). In CWH characters, the highest difference was shown by the effect of cropping system (C) by 68.79 %, while the effect of season (S) by 0.07 %, genotype (G) by 5.58 %, and GEIs (*G* × *S* × C) by 4.31 %. Then in the GY character, the highest difference was shown by the effect of planting system (C) of 70.00 %, while the effect of season (S) was 5.26 %, genotype (G) was 1.87 %, and the interaction of GEIs (*G* × *S* × C) was 5.44 %.

**Fig. 1:**
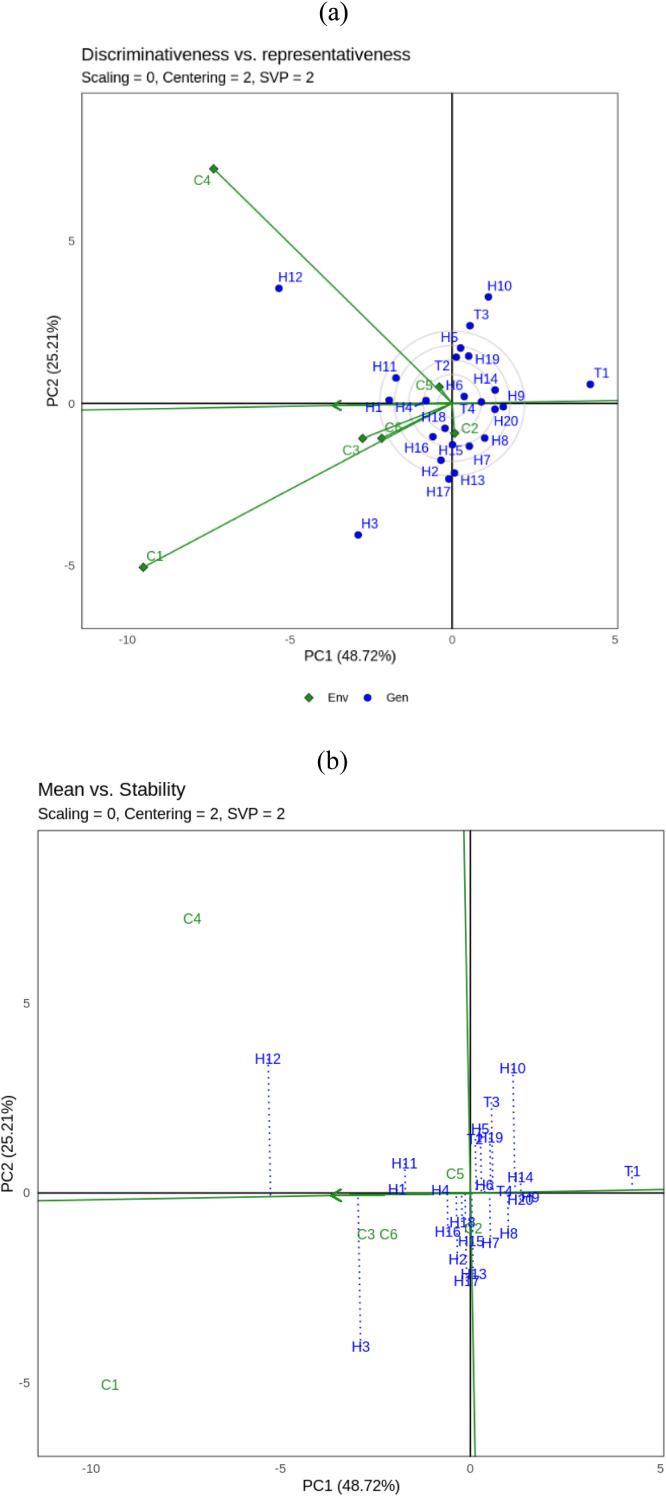

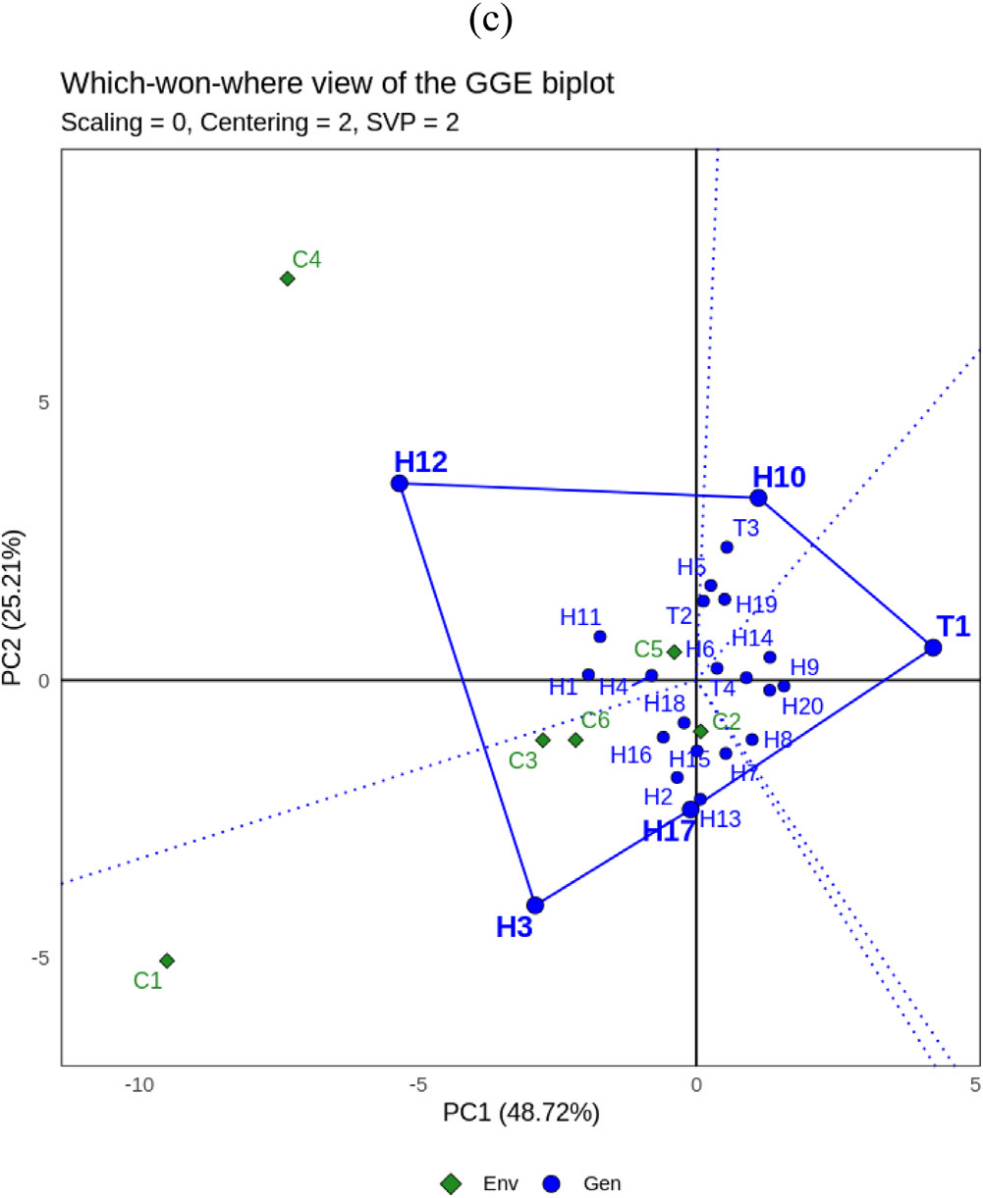
GGE biplot for biomass (CWH) of 24 maize hybrids under three different cropping system: a. **‘**Discriminativeness vs representativeness’, b.**‘**Mean vs stability’, c. **‘**Which won where’. Genotype code see Table.

**Fig. 2:**
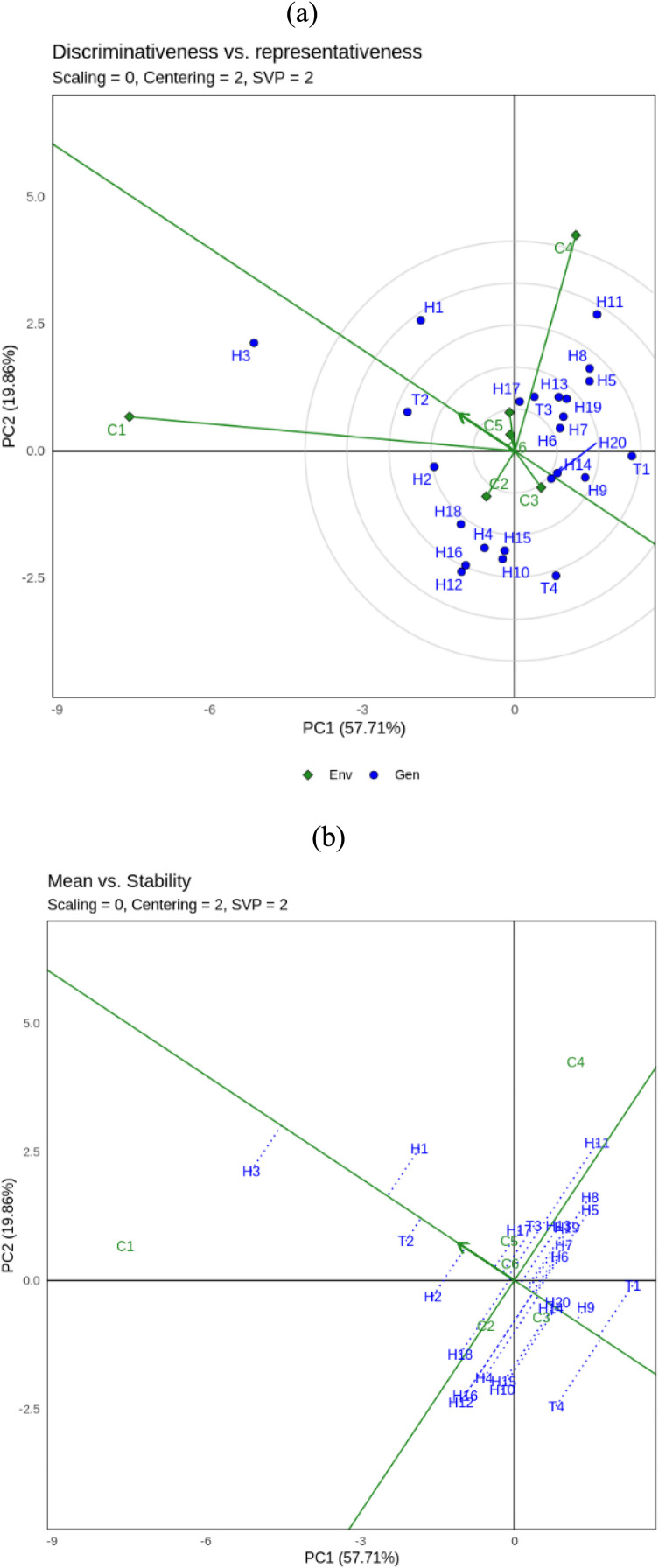

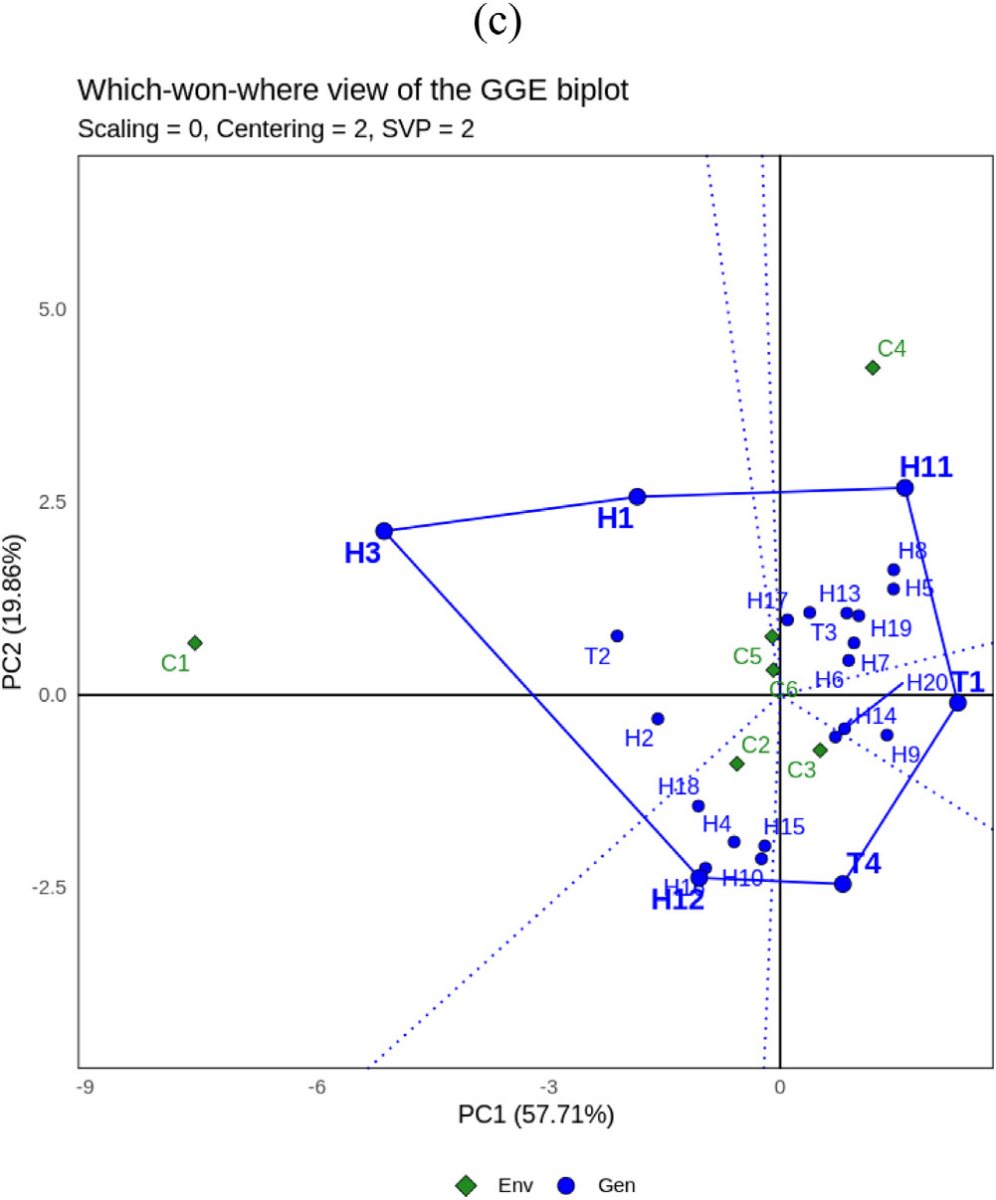
GGE biplot for yield (GY) of 24 maize hybrids under three different cropping system: a. **‘**Discriminativeness vs representativeness’, b.**‘**Mean vs stability’, c. **‘**Which won where’. Genotype code see Table.

Genotype and Genotype x Environment (GGE) Biplot for three cropping systems in two seasons divided into Environment 1 (C1/maize sole crop season 1), Environment 2 (C2/maize + soybean intercropping season 1), Environment 3 (C3/maize + sweet potato season 1),

Environment 4 (C4/maize single crop season 2), Environment 5 (C5/maize + soybean intercropping season 2) and Environment 6 (C6/maize + sweet potato season 2). Visualization of biomass stability (CWH) of hybrid maize using GGE biplot analysis is presented in Fig. 1a, b and c, while yield (GY) is presented in Fig. 2a, b and c.

GGE biplot analysis of 24 hybrid maize showed that PC1 and PC2 contributed 48.72 % and 25.21 % respectively to the total variation of hybrid maize yield (Fig. 1a, b and c). Fig. 1a shows the “Discriminativeness vs. representativeness” of GGE biplots for biomass characters. The vector length of each location shows differences. C2, C3, C5 and C6 are type I environments and should not be used as test environments because they have short vectors. While C1 is a suitable environment (type II) because it has the longest vector and a small abscissa angle, this environment tends to have high results and is quite representative for all environments so it is a suitable environment for selecting superior hybrids and as a testing environment. C4 as type III which has a long vector and large abscissa angle cannot be used to select superior genotypes, but can be used to select genotypes that are adaptive or specialized in a particular environment.

Fig. 1b shows the “mean vs stability” of the GGE biplot for biomass characters. Ideal points are located and point to the left of the Y-axis. Nine hybrids were identified in the tested maize population that yielded more than the overall average (left of the Y-axis), namely H12, H3, H1, H11, H4, H16, H2, H18 and H17. In comparison, the other 15 genotypes yielded less than the overall average (right side of the Y-axis). Genotypes H1, H11, H4 and H16 were the most stable hybrids (closest to the X-axis), while genotypes H12 and H3 were unstable.

Fig. 1c shows the “Which won where” view of the GGE biplot for biomass. The analysis shows that the cropping system environment is divided into five sectors. The peak hybrid in each sector is called a vertex indicating that the genotype had the highest yield in each environment within that sector. Sectors consisting of more than one environment form a mega-environment. For example, the first sector is a mega-environment consisting of C1, C2, C3 and C6 with H3 and H17 occupying the vertices. Then the second sector has a mega-environment consisting of C4 and C5 with H12 occupying the vertex. While the other sector consists of H10 and T1 which occupy the top of each sector, but there is no environment in the sector. Therefore, in this study, the ideal genotypes for biomass characters based on which won where GGE biplot are H3, H17 and H12.

Fig. 2a shows the “Discriminativeness vs representativeness” of the GGE biplot for yield characters. C2, C3, C5 and C6 are type I environments and should not be used as testing environments because they have short vector distances that do not represent the tested maize hybrids. Whereas C1 is a suitable environment (type II) due to its strong discriminative and representative properties shown from the vectors that have a small angle to the abscissa and long distance so that this environment can be used to select superior genotypes. C4 is included in the type III environment so that it can be used to select genotypes that are adaptive or specialized in a particular environment.

The ‘Mean vs Stability’ biplot is presented in Fig. 2b. Eight maize hybrids were identified as yielding more than the overall mean and located to the left of the Y-axis, namely H3, H1, T2, H2, H17, T3, H11 and H18. However, the other 16 genotypes are located to the right of the Y-axis and have yields less than the overall average. Genotypes H3, H1, T2 and H2 were the most stable hybrids and were closest to the X-axis, while H11 and H18 were unstable. T2 is the ideal genotype and is close to the ideal point and the X-axis, so it has high yield performance (above the overall average yield) in various sole-cropping and multiple-cropping environments.

Fig. 2c shows the “Which won where” GGE biplot for yield. The analysis shows that the cropping systems environment is divided into six sectors. There is one sector that consists of more than one environment, forming a mega-environment, namely the first sector consisting of C1, C5 and C6 with H3 and H1 as vertices. The second sector has C4 and H11 as vertices. The next sector is C2 with vertex H12. Then the last sector that has an environment consists of C3 with T4 occupying the vertex. While the other sector consists of T1 which occupies the vertex, but there is no environment in the sector. Thus, in this study the ideal genotypes for biomass characters based on which won where GGE biplot are H3, H11, H11 and H12.

**Fig. 3:**
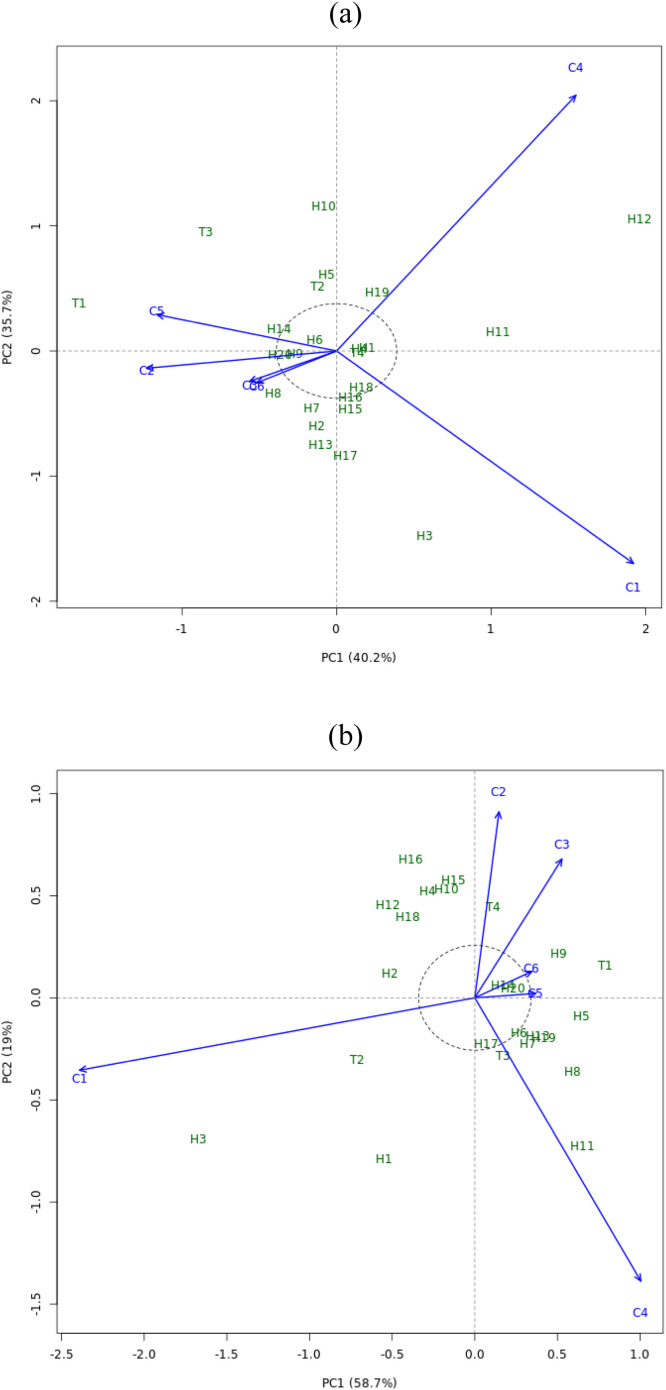
AMMI Biplot of 24 maize hybrids under three different cropping system: a. Biomass (CWH); b. Yield (GY). Genotype code see Table.

The AMMI2 biplots for biomass and yield are presented in Fig. 3a and b which show that there are genotypes that are stable in all test environments and there are specific genotypes in certain environments. In Fig 3a, the widely adapted genotypes based on biomass characteristics were T4, H4, H1, H6, H9, H18 and H20. H14 was found to have environment-specific adaptation in C5 and H19 adapted in C4. Based on the seed results in Fig. 3b, H13, H20 and H17 are widely adapted in all environments. While T4 has environment-specific adaptation in C2.

High yield stable genotypes on biomass are shown in Table 2. The ranking of genotypes with the highest biomass (rCWH) are H12, H3, H1, T4 and H4. While the ranking of stable genotypes (rASV) are T4, H4, H6, H1 and H9. The ranking of stable genotypes with high biomass (rGSI) are H1, H4, H16, H18 and H6.

**Table 2:** High-stable genotype for CWH of 24 maize hybrids under three cropping system.

Gen	CWH	rCWH	ASV	rASV	GSI	rGSI
H1	8.756975	3	0.2277562	4	7	1
H2	7.79801	6	0.6156969	16	22	7
H3	8.981728	2	1.6107813	22	24	9
H4	7.900556	5	0.1651713	2	7	1
H5	7.339649	12	0.6140148	15	27	13
H6	7.244609	15	0.1818535	3	18	5
H7	7.129239	16	0.485574	11	27	13
H8	7.067195	17	0.5711775	14	31	20
H9	6.516049	23	0.3053621	5	28	17
H10	6.740432	20	1.1629429	19	39	23
H11	7.611432	8	1.1809348	20	28	17
H12	9.624568	1	2.4395253	24	25	11
H13	7.401204	10	0.7551079	17	27	13
H14	6.833395	18	0.456236	9	27	13
H15	7.279993	14	0.475011	10	24	9
H16	7.763774	7	0.3834887	7	14	3
H17	7.337333	13	0.8336439	18	31	20
H18	7.390317	11	0.3435281	6	17	4
H19	6.760216	19	0.5573242	13	32	22
H20	6.733796	21	0.4081656	8	29	19
T1	5.936111	24	1.908563	23	47	24
T2	7.517938	9	0.5359845	12	21	6
T3	8.091738	4	1.3465013	21	25	11
T4	6.548452	22	0.1526591	1	23	8

CWH = cob weight with husk (biomass); rCWH = rank of CWH; ASV = AMMI stability index; rASV = rank of ASV; GSI = genotype stability index; rGSI = rank of GSI.

High yield stable genotypes on seed yield are shown in Table 3. The highest yielding genotypes (rGY) were H3, H1, H2, T2 and H5. Ranked stable genotypes (rASV) were H17, H14, T4, T3 and H15. Ranked stable genotypes with high yield (rGSI) were H17, T3, H13, H2, H5.

**Table 3:** High-stable genotype for GY of 24 maize hybrids under three cropping system.

Gen	GY	rGY	ASV	rASV	GSI	rGSI
H1	4.794568	2	1.85909	18	20	5
H2	4.712901	3	1.5837208	15	18	4
H3	5.287778	1	5.2063954	24	25	12
H4	4.286235	16	1.0168983	9	25	12
H5	4.52463	5	1.9948371	20	25	12
H6	4.073333	21	0.8361593	8	29	19
H7	4.173889	18	1.0237023	10	28	18
H8	4.349753	12	1.8637358	19	31	21
H9	4.308518	13	1.5853866	16	29	19
H10	4.230926	17	0.745642	7	24	10
H11	4.303364	14	2.1327229	21	35	22
H12	4.110124	19	1.6735872	17	36	23
H13	4.465864	6	1.197241	11	17	3
H14	4.04963	22	0.5389847	2	24	10
H15	4.287984	15	0.6993901	5	20	5
H16	4.424321	8	1.377584	14	22	8
H17	4.399321	9	0.3045344	1	10	1
H18	4.464198	7	1.3022106	13	20	5
H19	4.377037	10	1.3002327	12	22	8
H20	4.077346	20	0.7095017	6	26	15
T1	4.021049	23	2.4462294	23	46	24
T2	4.684568	4	2.2056537	22	26	15
T3	4.370926	11	0.5914402	4	15	2
T4	3.849568	24	0.5650185	3	27	17

GY = Grain Yield; rCWH = rank of GY; ASV = AMMI stability index; rASV = rank of ASV; GSI = genotype stability index; rGSI = rank of GSI.

Land equivalent ratio (LER) of maize+soybean (IC1) and maize+sweet potato (IC2) intercropping systems in season 1 and season 2 for biomass and yield are presented in Table 4. LER of biomass in IC1 in season 1 for all genotypes showed favorable criteria (LER >1) compared to planting in a single maize cropping system, in season 2 only two genotypes showed less favorable, namely H15 and H16. Biomass LER in IC2 in season 1 had four genotypes that showed favorable criteria, namely H7, H20, T1 and T3, as well as in season 2, namely H3, H8, H18 and T4.

**Table 4:** LER analysis for CWH and GY of 24 maize hybrids under two intercropping and two seasons.

**Gen**	**CWH**				**GY**			
	**IC1**		**IC2**		**IC1**		**IC2**	
	Season 1	Season 2	Season 1	Season 2	Season 1	Season 2	Season 1	Season 2
H1	1.25	1.37	0.96	0.94	1.08	1.41	0.63	0.79
H2	1.33	1.34	0.88	0.78	1.28	1.3	0.75	0.82
H3	1.22	1.43	0.73	1.17	1.16	1.23	0.52	1.02
H4	1.31	1.11	0.92	0.84	1.37	1.08	0.73	0.96
H5	1.36	1.25	0.78	0.72	1.57	1.15	0.73	0.75
H6	1.34	1.3	0.9	0.75	1.42	1.27	0.56	0.72
H7	1.39	1.27	1.05	0.93	1.37	1.19	1.01	0.84
H8	1.32	1.44	0.7	1.07	1.32	1.37	0.65	0.9
H9	1.48	1.42	0.84	0.95	1.36	1.5	1.03	1.11
H10	1.44	1.17	0.8	0.84	1.32	1.61	0.68	1.16
H11	1.07	1.03	0.64	0.84	1.24	1.01	0.74	0.81
H12	1.28	1.14	0.67	0.79	1.3	1.55	0.6	1.01
H13	1.29	1.43	0.81	0.96	1.32	1.43	0.86	0.75
H14	1.51	1.2	0.83	0.84	1.4	1.15	0.76	0.91
H15	1.34	0.85	0.81	0.76	1.4	1.06	0.78	0.68
H16	1.39	0.92	0.89	0.63	1.38	1.06	0.77	0.68
H17	1.2	1.05	0.86	0.75	1.34	0.9	0.72	0.64
H18	1.25	1.52	0.98	1.06	1.29	1.52	0.91	1.22
H19	1.25	1.39	0.72	0.8	1.29	1.46	0.8	0.89
H20	1.34	1.16	1.05	0.74	1.36	1.07	0.87	0.81
T1	1.98	1.21	1.23	0.65	1.66	0.96	0.88	0.57
T2	1.33	1.31	0.72	0.93	1.23	1.27	0.5	1.08
T3	1.54	1.65	1.01	0.88	1.32	1.53	0.68	0.7
T4	1.22	1.12	0.64	1.05	1.16	1.19	0.87	1.23

IC1 = maize+soybean intercropping; and IC2 = maize+sweet potato intercropping.

Furthermore, the LER for seed yield in IC1 in season 1 all genotypes showed favorable criteria (LER>1) compared to planting in a single maize cropping system, while in season 2 only two genotypes showed less favorable criteria, namely genotypes H17 and T1. Then the LER of seed yield in IC2 in season 1 only two genotypes showed favorable criteria, namely H9 and H7, while in season 2 there were 7 genotypes that showed favorable criteria, namely T4, H18, H10, H9, T2, H3 and H12. Furthermore, the ranking of genotypes based on LER for seed yield in IC1 in season 1 was T1, H5, H6, H14 and H15, while in season 2 it was H10, H12, T3, H18 and H9. Then the LER rankings in IC2 in season 1 were H9 and H7, while in season 2 they were T1, H18, H10, H9 and T2.

## 4 Experimental Design, Materials and Methods

The field evaluation was done in Arjasari, West Java, Indonesia, for two seasons in three different cropping systems, namely: maize sole cropping, maize+soybean intercropping and maize+sweet potato intercropping. Data of seasons, rainfall,temperature, and type of agroclimate in Arjasari is presented in Table 5. The evaluation applied a randomized completed block design with three replications. The hybrids were planted in a 3 × 3 m plot with a spacing of 0.75 × 0.2 m. Data were gathered during harvest, 93 days following planting, using the Descriptor for Maize [8]. Planting and harvesting were done manually. Each plot composed of 60 plants for sole-cropping and 30 for intercropping. The yield in each plot was converted into tons ha-^1^. The data gathered were vegetative and yield traits. The traits evaluated were plant weight and cob weight with husks (CWH) for biomass and grain yield (GY) for yield. Data collection has been adjusted to match the maize descriptor [8].

**Table 5:** Seasons, rainfall,temperature, agroclimate in arjasari.

Seasons	Rainfall (mm)	Temperature (°C)			Agroclimate
			Min.	Max.	Avg.
**Season 1** (Rainy to dry season)	283,2	18,2	32,2	25,7	AII2. Wet; ; dry month per year 3–7; wet month 5–9; crop index 2; pH 6; ultisol type of soil.
**Season 2** (Dry to rainy season)	237,3	19,6	30,4	24,2	

The study used 20 maize hybrids bred by the Plant Breeding Lab, at Padjadjaran University's Faculty of Agriculture (UNPAD) and four commercial hybrids as check varieties. The hybrids have a broad genetic origin (Table 6) [9].

**Table 6:** The maize hybrid materials used in the experiment.

Code	Parental			Pedigree
	Female		Male	
H1	DR 4	×	MDR 7.2.3	Female is downy mildew resistant (DMR) line, male is mutant line
H2	DR 4	×	MDR 16.6.14	Female is DMR line, male is mutant line
H3	DR 5	×	MDR 18.8.1	Female is DMR line, male is mutant line
H4	DR 6	×	DR 7	Female and male are DMR lines
H5	DR 7	×	DR 8	Female and male are DMR lines
H6	DR 8	×	MDR 18.8.1	Female is DMR line, male is mutant line
H7	DR 8	×	DR 9	Female and male are DMR lines
H8	DR 8	×	MDR 1.1.3	Female is DMR line, male is mutant line
H9	DR 10	×	MDR 9.1.3	Female is DMR line, male is mutant line
H10	DR 11	×	DR 16	Female is DMR line, male is high protein line
H11	DR 14	×	DR 18	Female is DMR line, male is high protein line
H12	DR 19	×	DR 20	Female and male are high protein lines
H13	MDR 3.1.4	×	MDR 18.5.1	Female and male are mutant lines
H14	MDR 3.1.2	×	MDR 153.14.1	Female and male are mutant lines
H15	MDR 7.4.3	×	DR 18	Female is mutant line, male is high protein line
H16	MDR 7.4.3	×	MDR 18.8.1	Female and male are mutant lines
H17	MDR 7.4.3	×	MDR 1.1.3	Female and male are mutant lines
H18	MDR 9.1.3	×	MDR 1.1.3	Female and male are mutant lines
H19	MDR 18.8.1	×	MDR 7.1.9	Female and male are mutant lines
H20	MDR 153.3.2	×	MDR 8.5.3	Female and male are mutant lines
T1	Bisi x			Hybrid commercial of Bisi Hybrid
T2	Bisi xy			Hybrid commercial of Bisi Hybrid
T3	Pertiwi x			Hybrid commercial of Pertiwi Hybrid
T4	NK xyz			Hybrid commercial of Monsanto Hybrid

PBtools software [10] was used to calculate the variance components of each trait. The combined ANOVA model for estimating GEIs included:

$$Y_{\text{ijkl}}=\mu +G_{i}+E_{j}+GE_{\text{ij}}+R_{k\left(i\right)}+B_{l\left(k\right)}+\varepsilon_{\text{ijkl}}$$

where Y_ijkl_ is the value of hybrids i in plot l, and the value of each replication in environment j;; μ is the grand mean yield; G_i_ is the effect of hybrid _i_; E_j_ is the effect of the environment j; GE_ij_ is an effect of genotype by environment interactions on hybrid i and environment j; R_k(j)_ is the effect of replication k on the location j; B_l(k)_ is the effect of replication k on plot l; and ε_ijkl_ is the error effects caused by mutant i in plot l and repeat k of environment j.

The genotype + genotype × environment (GGE) model defines variation due to genotype and genotype-environment interaction (GEI). [11] approximate the GGE biplot model using the following formula:

$$\;{\bar{Y}}_{mn}-\;\mu_{m}=\beta_{n}+\sum_{k=1}^{t}\lambda_{o}\alpha_{mo}\gamma_{no}+\varepsilon_{mn}$$

where Ῡ_mn_; μ_m_; β_n_; k; λ_o_; α_mo_ and γ_no_; ε_mn_ represent the appearance in environment ‘n’ from hybrid ‘m’; a total of average yield; the effect of environment ‘n’; the number of primer components; the singular value from primer component ‘o’; value of hybrid ‘m’ and environment ‘n’ for primer component ‘o’; and the hybrid ‘m’ error in environment ‘n’, respectively. The GGE biplot analysis was estimated using RStudio.

The stability of the AMMI model for evaluating maize hybrid yield is based on [12]:

$$Y_{ef}=\mu +Ge+E_{f}+\sum_{k=1}^{n}\left(\lambda_{g}\;\alpha_{eg}\;\gamma_{fg}\right)+\rho_{ef}$$

Where: Y_ef_ represent the yield performance of genotype eth in the environment f_th_, µ is the average of all yield performances from genotypes used, G_e_ is the genotype eth mean deviation, E_f_ is the environment f_th_ mean deviation, λ_k_ is the square root of the eigenvalue of the PCA axis g, α_eg_ and γ_fg_ are the PC score for PCA axis g of the genotype i_th_ and the environment f_th_, respectively, and ρ_ef_ is the residual.

AMMI stability value (ASV) is evaluated using the following formula [13]:

$$\text{ASV}=\;\sqrt{\frac{\text{SS}\;\text{IPCA}\;1}{\text{SS}\;\text{IPCA}\;2}{\left(\text{IPCA}\;1\;\text{Score}\right)}^{2}+{\left(\text{IPCA}\;2\;\text{Score}\right)}^{2}}$$

Where: The weights assigned to the IPCA 1 and IPCA 2 scores are ss IPCA1 and ss IPCA2, which are calculated by dividing ss IPCA 1 and ss IPCA 2. The AMMI analysis composes of two IPCA ratings for each genotype: IPCA 1 and IPCA 2. Low ASV scores determine maize hybrids that are stable in a variety of conditions.

The genotype stability index (GSI) was calculated using the hybrids’ ASV rank (rASV) and the rank of biomass and yield (rX) features in the three cropping systems using the following formula [1]:

GSI=rASV+rX

The land equivalent ratio (LER) is counted based on the formula of [4]:

$$\text{LER}=\;\sum_{i=1}^{a}\frac{IC_{i}}{C_{i}}$$

Where IC_i_ represent biomass or yield average each genotype of crop ‘i’ in intercropping; C_i_ is biomass or yield average each genotype of crop ‘i’ in sole-crop.

## Limitations

The field experiment is conducted in one location, therefore, need to confirm in another crop production area to get more additional data.

## Ethics Statement

The dataset described in this article does not involve any human subjects, animal experiments, or data collected from social media platforms.

## Credit Author Statement

**Mansyur:** Conceptualization, Data gathering, Validation, Methodology, Writing – draft. **Dedi Ruswandi:** Conceptualization, Soft ware, Supervision, Resources, Funding acquisition, Methodology, Project administration, Writing – review & editing.

## Data Availability

Mendeley Datadataset for stability of high biomass and yield in maize under normal and intercropping conditions based on biplot, genotype stability index and land equivalent ratio (Original data).
